# Effects of Changing pH, Incubation Time, and As(V) Competition, on F^−^ Retention on Soils, Natural Adsorbents, By-Products, and Waste Materials

**DOI:** 10.3389/fchem.2018.00051

**Published:** 2018-03-06

**Authors:** Ana Quintáns-Fondo, Vanesa Santás-Miguel, Juan C. Nóvoa-Muñoz, Manuel Arias-Estévez, María J. Fernández-Sanjurjo, Esperanza Álvarez-Rodríguez, Avelino Núñez-Delgado

**Affiliations:** ^1^Department of Soil Science and Agricultural Chemistry, Engineering Polytechnic School, Universidade de Santiago de Compostela, Lugo, Spain; ^2^Department of Plant Biology and Soil Science, Faculty of Sciences, University of Vigo, Ourense, Spain

**Keywords:** by-products, fluoride pollution, soils, sorption, wastes

## Abstract

The purpose of this work was to elucidate the repercussion of changing pH, incubation time and As(V) competition on fluoride (F^−^) sorption on forest and vineyard soil samples, pyritic, and granitic materials, as well as on the by-products pine sawdust, oak wood ash, mussel shell ash, fine and coarse mussel shell, and slate processing waste fines. To reach this end, the methodological approach was based on batch-type experiments. The results indicate that, for most materials, F^−^ sorption was very high at the start, but was clearly diminished when the pH value increased. However, oak wood ash and shell ash showed high F^−^ sorption even at alkaline pH, and pine sawdust showed low F^−^ sorption for any pH value. Specifically, F^−^ sorption was close to 100% for both ashes at pH < 6, and around 70% at pH 10, while for forest soil it was close to 90% at pH < 2, and around 60% at pH values near 8. Regarding the effect of incubation time on F^−^ sorption, it was very low for both soils, pyritic material, granitic material, and both kinds of ashes, as all of them showed very rapid F^−^ sorption from the start, with differences being lesser than 10% between sorption at 30 min and 1 month of incubation. However, sawdust and slate fines sorbed 20% of added F^−^ in 30 min, remaining constant up to 12 h, and doubling after 30 days. And finally, mussel shell sorbed 20% at 30 min, increasing to close to 60% when incubation time was 30 days. This means that some of the materials showed a first sorption phase characterized by rapid F^−^ sorption, and a slower sorption in a second phase. As regards the effect of the presence of As(V) on F^−^ sorption, it was almost negligible, indicating the absence of competition for sorption sites. In view of that all, these results could aid to appropriately manage soils and by-products when focusing on F^−^ removal, in circumstances where pH value changes, contact time vary from hours to days, and potential competition between F^−^ and As(V) could take place.

## Introduction

Fluoride (F^−^) is present in rocks, soil, air, water, and plants. While low intake of F^−^ can be beneficial for teeth in humans (preventing caries) and bone growth, excessive F^−^ concentrations can result in fluorosis and other adverse effects on human health, such as those regarding correct mental development in children, as previously shown by Oruc (2008), Yadav et al. (2009), Patel et al. (2014), and Yesilnacar et al. (2016). In fact, Chen et al. (2012) stated that fluorosis is becoming a global toxicological concern.

Msonda et al. (2007) indicated that F^−^ concentrations are usually lower than 0.3 mg L^−1^ in non-polluted waters, but concentrations higher than 1.0–1.5 mg L^−1^ are considered problematic (WHO, 2004). Fluoride concentration in groundwater can range from less than 1 to more than 35 mg L^−1^ (Maheshwari, 2006), depending on soil acidity, porosity, geology, chemical parameters, temperature, and other variables (Chaudhary et al., 2008).

Cronin et al. (2003) and Kumar et al. (2014) indicated that the geological substrate is of great importance regarding F^−^ concentration in soils, taking into account its presence in minerals such as apatite, topaz, cryolite, and fluorite. But other authors, such as Gago et al. (2002), Weinstein and Davison (2004), and Gago et al. (2014), have signaled that industrial sources are also important, especially aluminum and phosphate-fertilizer factories. In aluminum smelters, Al is produced by electrolysis of alumina (Al_2_O_3_) dissolved in molten cryolite (Na_3_AlF_6_), which causes the emission of fluoride (appearing as gaseous HF or in particulate form); and this F^−^ is considered as the highest-impact phytotoxic pollutant derived from the aluminum reduction process (Kumar and Rani, 2011). Furthermore, agriculture and forestry also affect soil F^−^ content, mainly due to some agrochemicals (Loganathan et al., 2001). Specifically, long-term phosphate fertilization on farmlands may cause very relevant F^−^ accumulation in soils (Loganathan et al., 2008; Kim et al., 2016), because F^−^ concentrations in P fertilizers are much higher (up to 150 times) than in soils (Stacey et al., 2010). Kalinic et al. (2005) reported that 300–500 mg kg^−1^ are normal values for total-F in soils, while, as commented by Brougham et al. (2013), concentrations higher than 500–600 mg kg^−1^ are indicative of F-rich minerals, industrial or agricultural pollution.

Brougham et al. (2013) also indicated that, even more important than total-F content, F availability in soils is very relevant, and it is highly dependent on factors such as pH and clay content, as well as on P, Ca, and Al concentrations. Authors such as Elrashidi and Lindsay (1986a,b), Khare et al. (2005) and Zhu et al. (2006) have remarked the high affinity of F^−^ for Al^3+^, with AlF_x_ complexes being the main F^−^ species in the soil solution of natural soils at pH 4–5.5 (Álvarez et al., 2002, 2003, 2005). In addition, these complexes are toxicologically relevant, acting as phosphate analogs for different enzymes (Strunecka et al., 2012). Fluoride is preferentially adsorbed by amorphous Al oxy-hydroxides, frequently found in acid soils (Zhu et al., 2006; Kaufhold et al., 2010; Gago et al., 2012, 2014). In fact, Arnesen and Krogstad (1998) detected maximal F^−^ adsorption on soils at pH 4.8–5.5, decreasing at pH values >5.5 due to the generation of negative charges (Barrow and Ellis, 1986; Gago et al., 2012, 2014). However, Wenzel and Blum (1992) found low F^−^ pollution risk in slightly acid soils, which increased in alkaline and strongly-acidic conditions.

Considering the situation of Galicia (NW Spain) as an example, F^−^ pollution is mainly related to aluminum smelter activities (a very relevant anthropogenic source for F^−^), and to P fertilization practices (due to F^−^ present in P fertilizers), which is a common practice in acidic soils, poor in nutrients (Gago et al., 2012, 2014).

Various F^−^ removal strategies have been investigated in the last decades (Raichur and Basu, 2001; Maheshwari, 2006), with growing research on the use of various agricultural by-products, as noted by Khalil (1996), Toles et al. (1998), or Wafwoyo et al. (1999), and more recently by Elizalde-González et al. (2008), and by Soleimani and Kaghazchi (2008). In addition, an increasing interest on waste recycling and valorization of by-products has been evidenced (Núñez-Delgado et al., 2015), and different bio-sorbents, such as pine bark, wood ash, or mussel shell, have been investigated, focusing on their potential to retain or remove cationic and/or anionic pollutants (see for example Fernández-Pazos et al., 2013; Ramírez-Pérez et al., 2013; Seco-Reigosa et al., 2013a,b; Osorio-López et al., 2014; Otero et al., 2015).

Regarding the effect of incubation time, previous studies have shown different velocities for F^−^ sorption on various materials. As an example, Peek and Volk (1985) found that sorption was rapid on the soils they studied, with 90% of the sorption occurring within 24 h. Tripathya et al. (2006) detected rapid F^−^ sorption (within 3 h) on impregnated alumina, whereas Tripathya and Raichur (2008) found two phases for F^−^ sorption on activated alumina (one fast, and another slower), and Bharali and Bhattacharyya (2015) indicated that the equilibrium time for F^−^ sorption was 60 min when using neem leaf powder as sorbent.

As regards competitive sorption, Ma et al. (2017) indicated that the concurrence of inorganic As and F^−^ in groundwater has been reported in many countries, with levels well above those set by World Health Organization as allowable maxima. Wu et al. (2007) found that increasing As concentrations added to a Fe-Al-Ce oxide did not affect F^−^ sorption, suggesting heterogeneous adsorption on the surface of the oxide. Ismail and AbdelKareem (2015) found that competing anions did not show significant repercussion on F^−^ removal capacity when using waste lamb or bones as sorbents, which could be due to the abundance of sorption and exchangeable sites on these materials. Jadhav et al. (2015) reviewed technologies allowing removal of As and F^−^ simultaneously, including adsorption, indicating that each technology has shortcomings and benefits. These last authors highlight the importance of the simultaneous removal of both F^−^ and As, concluding that it would be clearly interesting to develop and implement an hybrid and sustainable low-cost technology, which could be reached by means of extensive research. In addition, researching about competence for sorption sites among F^−^ and other anions than those related to As would be clearly interesting for future works.

In recent works we studied F^−^ sorption and desorption on different soil samples, wastes, by-products and waste mixtures (Quintáns-Fondo et al., 2016a), and on individual and amended materials (Quintáns-Fondo et al., 2016b). These different materials were: forest and vineyard soil samples, pyritic and granitic materials, pine sawdust, oak wood ash, mussel shell ash, fine and coarse mussel shell, and slate processing waste fines. They were selected for these previous works taking into account the following facts: (a) an aluminum facility in Galicia (NW Spain) is a source of F^−^ pollution affecting surrounding soils; (b) pyritic mine tailings and slate waste dumping sites are degraded areas subjected to restoration treatments, which implicate the addition of waste and by-products, such as pine sawdust, oak wood ash, mussel shell, or mussel shell ash, individually or as mixtures (Raichur and Basu, 2001; Quintáns-Fondo et al., 2016a) (c) these degraded areas also receive a variety of other waste and by-products, potentially including F^−^ and/or As(V) among pollutants. In these studies, we adjusted data to adsorption isotherms, and found overall promising results. However, the effects of changing pH, incubation time or competition with other anions were not studied.

Taking all that into account, in this research we focused on the effects of changing pH, incubation time, and As(V) competition, on F^−^ retention capacity on different soil samples, pyritic and granitic materials, as well as on different by-products: pine sawdust, oak wood ash, mussel shell ash, fine and coarse mussel shell, and slate processing waste fines. The results could be of aid in order to manage soils and by-products such as those here studied. Specifically, they would be interesting when focusing on F- retention/removal in the following circumstances: (a) when pH and contact time change, and (b) when F- and As(V) are present simultaneously in the solid or liquid media under investigation.

## Materials and methods

### Materials

We used: a forest soil sample, a vineyard soil sample, pyritic material, granitic material, fine (<1 mm), and coarse (0.5–3 mm) mussel shell, mussel shell calcination ash, oak wood ash, pine sawdust, and slate processing waste fines. These materials were described in previous papers by Seco-Reigosa et al. (2013a), Osorio-López et al. (2014), Seco-Reigosa et al. (2014, 2015), and Otero et al. (2015). See more details, as well as maps and tables corresponding to characterization of these materials, in Supplementary Material.

### Methods

#### F^−^ sorption for different pH values

Triplicate 1-g samples of each of the various soil samples, by-products and waste materials were added with 10 mL of solutions containing F^−^ at 100 mg L^−1^ (prepared from analytical grade KF, Panreac, Spain) and different concentrations of HNO_3_ (0.005, 0.05, and 0.1 M) or NaOH (0.005, 0.01, 0.02, 0.04, 0.08, and 0.1 M), also including 0.01 M NaNO_3_ as background electrolyte (HNO_3_, NaOH and NaNO_3_ from Panreac, Spain). Different control samples were constituted by each of the sorbents with 10 mL of solutions containing 0.01 M NaNO_3_ and F^−^ at 100 mg L^−1^, but without HNO_3_ or NaOH. All these samples were shaken (for 24 h), centrifuged (for 15 min at 4000 rpm, equivalent to 6,167 × g), and filtered (using acid-washed paper). The resulting liquid was analyzed for pH by means of a glass electrode (Crison, Spain) (Tan, 1996), and an ion-selective electrode to quantitatively determine F^−^ (measured after adding a total ionic strength adjuster and the TISAB IV buffer -Orion Research, Cambridge, USA). Sorbed F^−^ was calculated as the difference between the added-F^−^ concentration and the F^−^ concentration in the equilibrium solution.

#### F^−^ sorption for different incubation times

Triplicate 10-g samples corresponding to the soils, by-products and waste materials were added with 100 mL of a 0.01 M NaNO_3_ solution containing F^−^ at 100 mg L^−1^ (1:10 solid:solution ratio), maintaining the contact during 1 month (720 h). The resulting pH values at time zero were: forest soil 6.11, vineyard soil 5.54, pyritic material 4.67, granitic material 6.06, fine mussel shell 8.77, coarse mussel shell 9.06, mussel shell calcination ash 10.16, oak wood ash 11.02, pine sawdust 4.74, and slate processing waste fines 7.09.

Aliquots (5 mL each) were taken at different incubation times: 0.5, 1, 2, 4, 8, 12, 24, 168, and 720 h, then the suspensions were centrifuged (for 15 min at 4000 rpm (6,167 × g)) and filtered using acid-washed paper. The resulting filtrate was analyzed for F^−^ as indicated above.

#### F^−^ sorption in competition with As(V)

Triplicate 3-g samples (<2 mm fraction) of each of the soils, by-products and waste materials were added simultaneously with F^−^ and As(V): specifically, 30 mL of 0.01 M NaNO_3_ solutions containing in all cases the same F^−^ concentration (3 mmol L^−1^), and different As(V) concentrations (0, 0.5, 1.5, 3, and 6 mmol L^−1^), prepared from analytical grade Na_2_HAsO_4_·7H2O (Panreac, Spain).

In parallel, other samples were added with 3 mmol L^−1^ of As(V) in all cases, and, simultaneously, with different F^−^ concentrations (0, 0.5, 1.5, 3, and 6 mmol L^−1^).

The resulting suspensions were shaken (for 24 h), centrifuged (at 4000 rpm for 15 min, 6,167 × g), and filtered through acid-washed paper. In the equilibrium dissolutions, pH and F^−^ were determined as indicated above, whereas As was quantified by means of ICP-mass (820-NS, Varian, USA). Sorbed As and F^−^ were calculated as the difference between added As(V) and F^−^, and As and F remaining in the equilibrium solution. As and F^−^ were determined by triplicate in all samples.

### Data analyses

Statistical analyses (mainly descriptive statistics, specifically average values, standard deviation, and coefficients of variation, as well as test for normality and analysis of variance when applicable) were performed by means of SPSS 19.0 (IBM, USA). When applicable, significance of statistical differences was considered at the level P 0.005.

## Results and discussion

### F^−^ sorption for different pH values

**Figure 1:**
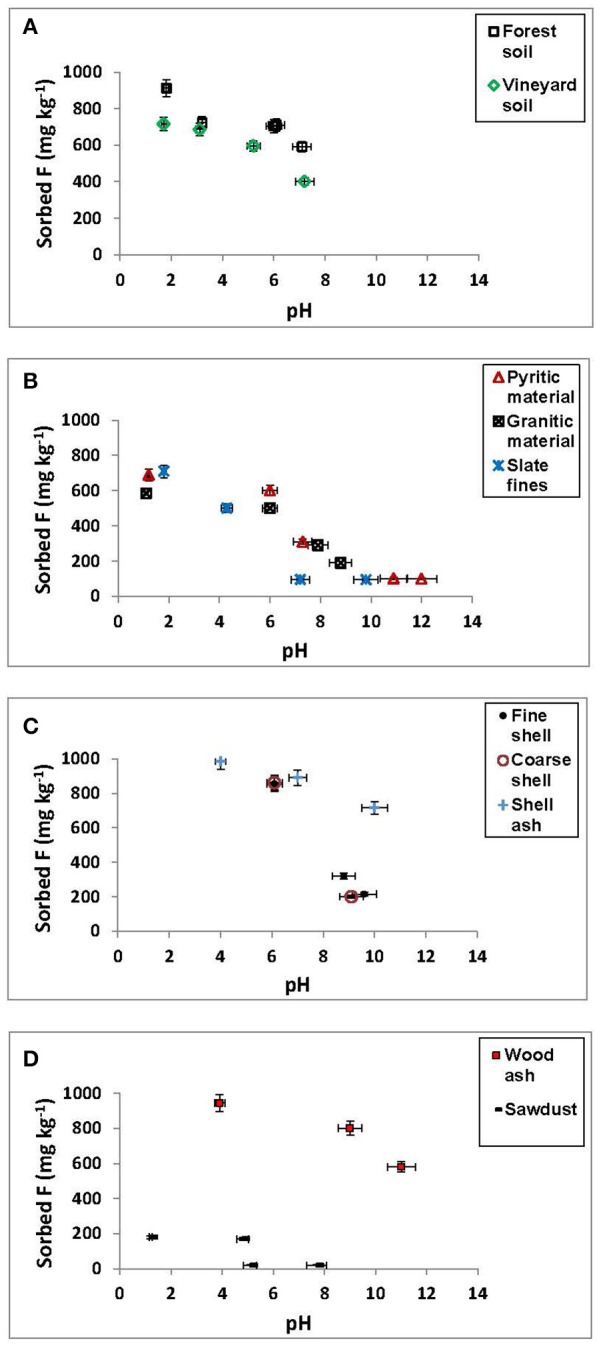
Sorption results (mg kg^−1^) for F^−^ on the soils, by-products and waste materials studied, at different pH values. Average values (three replicates), with coefficients of variation <5%.

Figure 1 shows that F^−^ sorption clearly decreased for most materials when pH increased, especially, and significantly, from pH 6, although shell ash and wood ash maintained high sorption up to alkaline pHs (Figures 1C,D), and sawdust showed low F^−^ sorption at any pH value (Figure 1D).

F^−^ sorption was close to 100% for both ashes at pH < 6 (Figures 1C,D), and at pH 10 was still higher than 70% for shell ash. F^−^ sorption on the forest soil sample was also high, close to 90% at pH < 2, and around 60% at pH values close to 8 (Figure 1A). Mussel shells sorbed about 90% of added F^−^ at pH < 7, showing a clear and significant decrease at alkaline pH (Figure 1C).

It must be taken into account that, at acid pH, the non-crystalline or low-crystallinity components present positive charge, which allows F^−^ sorption by means of electrostatic interactions, forming outer-sphere complexes (Valdivieso et al., 2006), or by exchange between F^−^ and OH^−^ groups, giving inner-sphere complexes, as indicated by Simard and Lafrance (1996), and by Shin and Han (2004). At these pH values, organic matter compounds can sorb F^−^ by means of H bindings or -${\text{NH}}_{3}^{+}$ groups. At pH values close to 6, sorption on variable-charge components negatively charged can take place through cationic bridges, but it is also possible that the formation of positively charged Al-F complexes (${\text{AlF}}_{2}^{+}$, AlF^2+^) takes place, those being very abundant in solution at pH between 5 and 6 (Álvarez et al., 2002, 2005), and these complexes can sorb directly on negatively charged components. Tang et al. (2009) found a similar pH range (from 3 to 6) for maximum F^−^ sorption on Fe hydroxides, which they attributed to the formation of HF species (which are more difficult to sorb) at pH < 3, and to the de-protonation of the sorbent surfaces at pH >6.

Wood ash and shell ash maintained a high F^−^ removal capacity even at alkaline pH, since the non-crystalline components are negatively charged, facilitating that sorption can take place through a cationic bridge involving Ca. Also, fluorite (CaF_2_) precipitation can occur (Fluhler et al., 1982; Elrashidi and Lindsay, 1986a,b). In this regard, Turner et al. (2005) explained F^−^ sorption on calcite as a combination of sorption reactions across the surface, and of mineral precipitation (in the form of fluorite) in the edges, where Ca^2+^ dissolution is greater.

### F^−^ sorption at different incubation times

**Figure 2:**
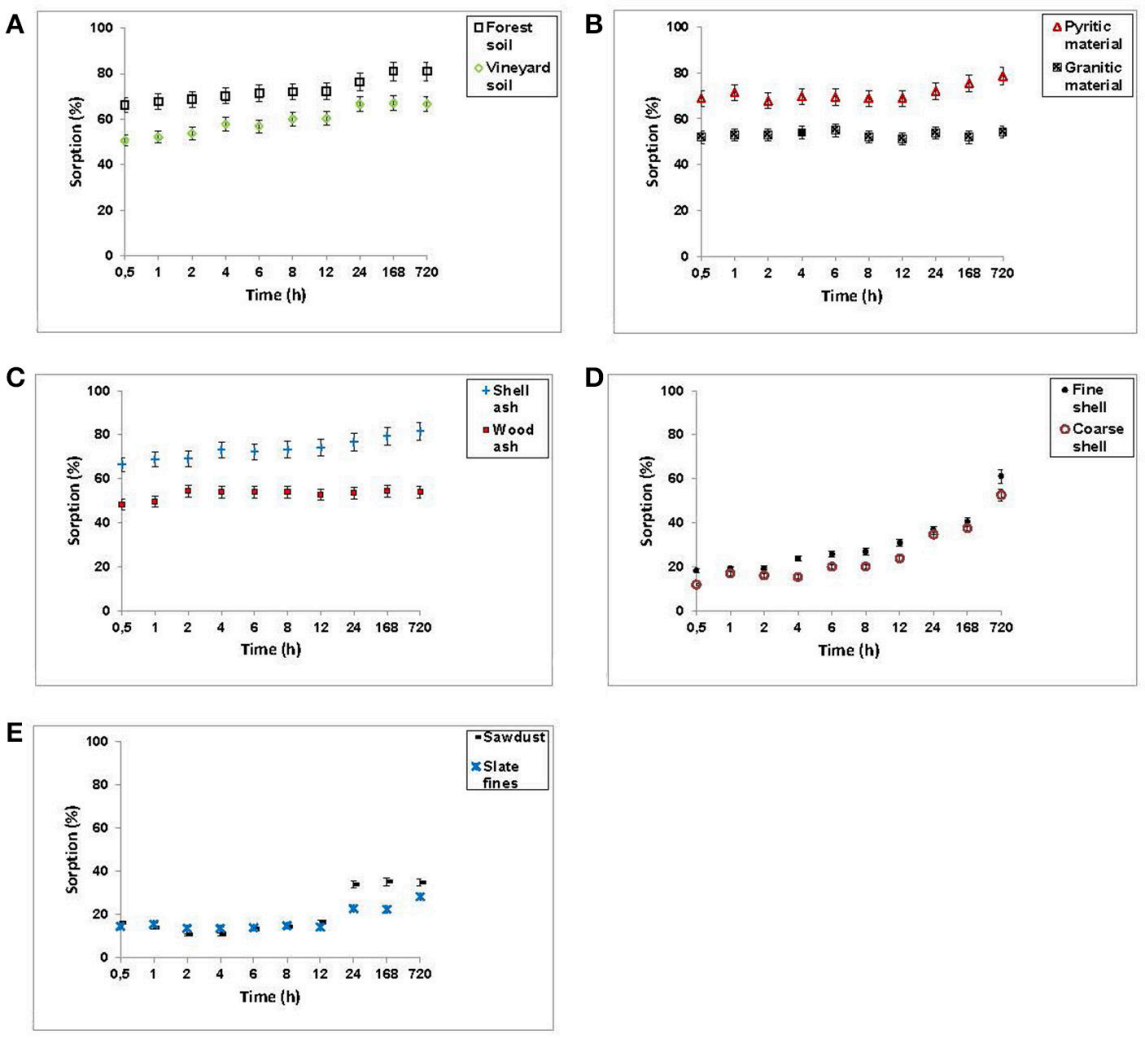
Sorption results (expressed as %) for F^−^ on the soils, by-products and waste materials studied, at different incubation times. Average values (three replicates), with coefficients of variation <5%.

Figure 2 shows F^−^ retention (in percentage) for the various soils, by-products and waste materials for different incubation times.

In view of the results, three groups of materials can be considered. The first group (soil samples, pyritic material, granitic material, and both kinds of ashes) was characterized by rapid F^−^ sorption, with differences being lesser than 10% between sorption at 30 min and 1 month of incubation (Figures 2A–C). The initial rapid sorption on these materials can be due to functional groups and surface sites very active in F^−^ sorption (Mohan and Karthikeyan, 1997). Tripathya et al. (2006) obtained 92% sorption of the added F^−^ in just 3 h using impregnated alumina, indicating that this rapid sorption was probably due to diffusion processes in the pores of the sorbent surface.

In a second group, sawdust and slate fines sorbed 20% of added F^−^ in 30 min, this percentage remaining constant up to 12 h, and doubling after 30 days of incubation (Figure 2E).

And finally, in a third group, mussel shells sorbed 20% of added F^−^ at 30 min, increasing to percentages close to 60% when incubation time was 30 days, with the largest increase taking place between days 7 and 30 (Figure 2D).

Therefore, in the last two groups of materials, at least two phases were evidenced during the sorption process: a first one requiring no more than 30 min, and a second one resulting in much higher F^−^ sorption after 30 days of incubation. Meenakshi and Viswanathan (2007) noted that when retention is very fast (in <40 min), it is due to the occurrence of a process of ion exchange, whereas when sorption is clearly increased after that time, it is indicative of surface sorption processes. Studying F^−^ sorption on activated alumina, Tripathya and Raichur (2008) detected two stages, indicating that such behavior can be explained by surface sorption phenomena as well as by intra-particle diffusion. Srimurali et al. (1997) indicate that, initially, all sorption sites are vacant, and the gradient of the solute concentration is high, and in a second phase process slow down due to the decrease of sorption sites.

### F^−^ sorption in competition with As(V)

**Figure 3:**
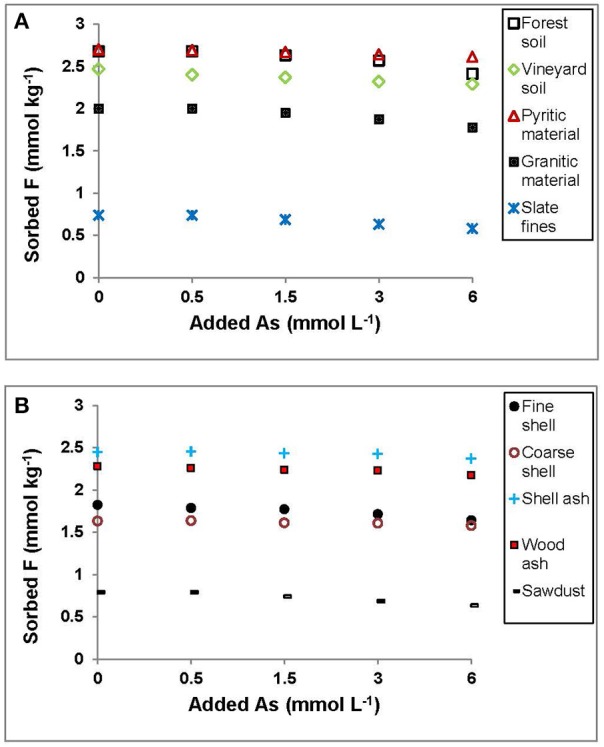
Sorption results (mmol kg^−1^) for F^−^ on the soils, by-products and waste materials studied, when the concentration of F^−^added is always 3 mmol L^−1^ and added As(V) is increased from 0 to 6 mmol L^−1^. Average values (three replicates), with coefficients of variation <5%.

Figure 3 shows that F^−^ sorption decreased very slightly when the amount of added As(V) increased, thus suggesting the absence of competition for sorption sites in the materials assayed. Similar results were found by Wu et al. (2007), who indicated the possible existence of heterogeneous sorption sites on the surface of the sorbent, causing no interferences with F^−^ sorption even when a high As(V) concentration was added.

**Figure 4:**
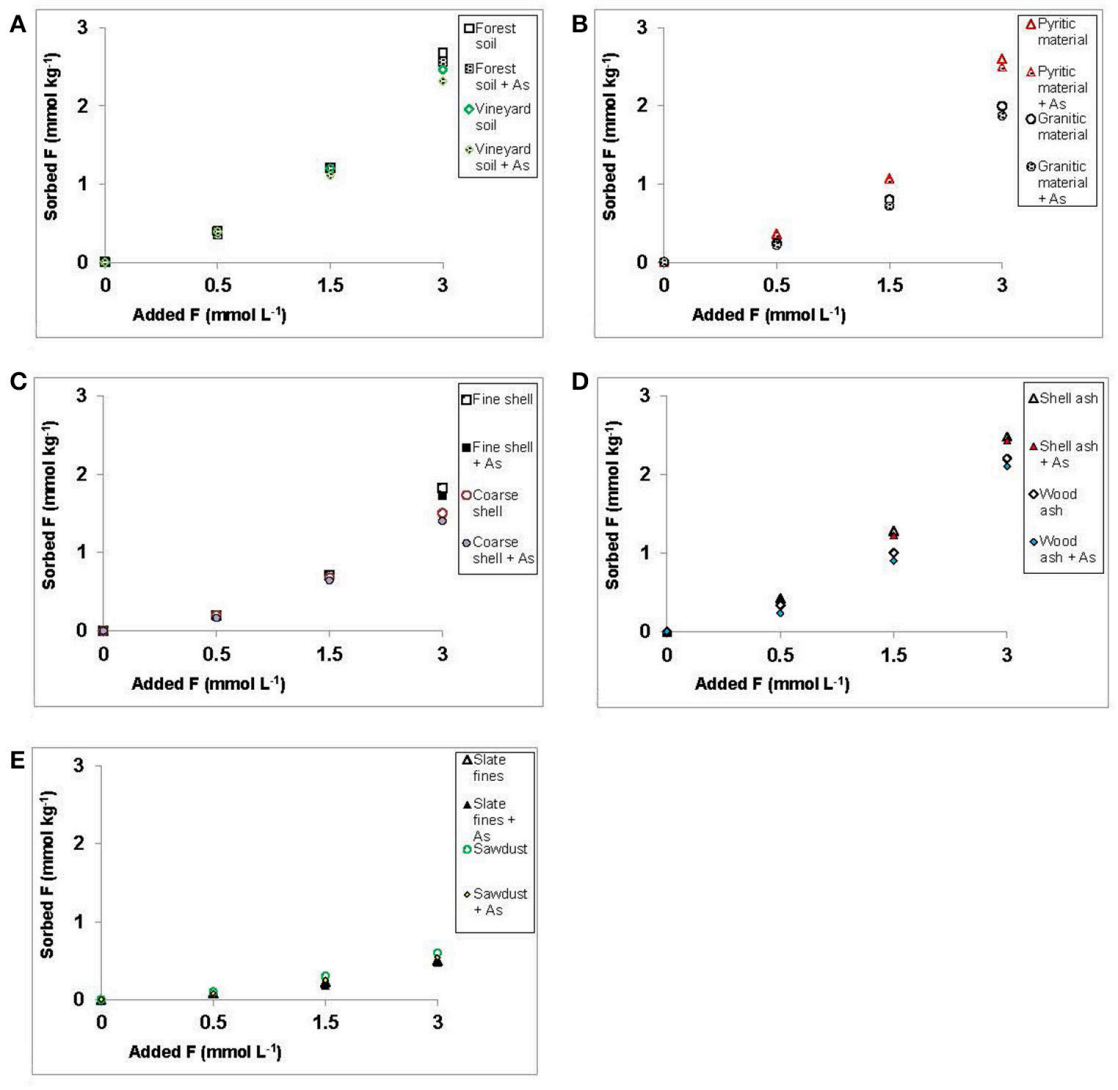
Sorption results (mmol kg^−1^) for F^−^ on the soils, by-products and waste materials studied, for increasing F^−^ concentrations added, both in absence of As(V) or when 3 mmol L^−1^ of As(V) are added. Average values (three replicates), with coefficients of variation <5%.

Figure 4 shows that the presence or absence of 3 mmol of As(V) did not affect sorption of increasing concentrations of F^−^ added to the various soil samples, by-products and waste materials studied. Dadwhal et al. (2011) found that competition between F^−^ and As(V) for sorption on an oxide-based material was very low. Liu et al. (2012) did not find competitive effect between As(V) and F^−^ for sorption sites on Fe, Al, and Fe-Al oxi-hydroxides, concluding that Fe oxi-hydroxides have a high As(V)-sorption potential, but very low F^−^ sorption capacity. In addition, these authors indicate that Al oxi-hydroxides can sorb both anions (but the efficiency largely depends on the pH, and there is a competition between both anions for sorption sites), and Fe-Al oxi-hydroxides have a great capacity to adsorb both anions in a wide range of pH. Notably, all soil samples, by-products and waste materials tested in the present study have relevant concentrations of Fe and Al oxy-hydroxides (see Supplementary Material).

## Conclusions

In this study we tested the effect of pH, incubation time and As(V) competition on F^−^ sorption, using a forest soil sample, a vineyard soil sample, pyritic material, granitic material, fine mussel shell, coarse mussel shell, mussel shell ash, oak wood ash, pine sawdust, and slate processing fines. As regards the effect of pH, F^−^ sorption clearly diminished in most materials when pH increased, notably from pH 6, although wood ash and shell ash maintained high F^−^ sorption even at alkaline pH, and pine sawdust showed low F^−^ sorption at any pH value. Incubation time (from 30 min to 30 days) did not affect substantially to both soil samples, pyritic material, granitic material and both kinds of ashes, which showed very rapid F^−^ sorption, whereas the other materials (slate fines, sawdust, and both kinds of mussel shell) showed a first phase of rapid F^−^ sorption and a second phase characterized by slower sorption. In addition, no remarkable competition was detected between F^−^ and As(V) for sorption sites. These results could aid to correctly manage soils, by-products and waste materials when focusing on F^−^ removal in circumstances where pH changes, contact time may vary from hours to days, and F^−^ and As(V) are present simultaneously in the affected media. Further future research would aid to deepen understand each of the relevant processes taking place during the sorption/desorption phases, and for each of the circumstances here considered. For instance, complementary research and determinations would be needed in order to elucidate actual adsorption sites involved in fluoride removal for the variety of sorbent materials investigated.

## Author contributions

JN-M, MA-E, MF-S, EÁ-R, and AN-D conceived and designed the study. AQ-F, VS-M, and EÁ-R carried out the experiments. AQ-F, VS-M, JN-M, MA-E, MF-S, EÁ-R, and AN-D discussed the results of the experiments, wrote, and revised the manuscript.

### Conflict of interest statement

The authors declare that the research was conducted in the absence of any commercial or financial relationships that could be construed as a potential conflict of interest.
