# Supplementary material for: Effects of Changing pH, Incubation Time, and As(V) Competition, on F− Retention on Soils, Natural Adsorbents, By-Products, and Waste Materials
Source: Front Chem. 2018 Mar 6;6:51. doi: 10.3389/fchem.2018.00051 (PMC5845531; doi:10.3389/fchem.2018.00051)
Supplement: Supplementary file 1 [file Table1.DOCX]

Supplementary Material

Fluoride retention on soil samples and by-products: Effects of pH, incubation time and As(V) competition

Ana Quintáns-Fondo^1^, Vanesa Santás-Miguel^2^, Juan Carlos Nóvoa-Muñoz^2^, Manuel Arias-Estévez^2^, María J. Fernández-Sanjurjo^1^, Esperanza Álvarez-Rodríguez^1^ and Avelino Núñez-Delgado^1*^

^1^Department of Soil Science and Agricultural Chemistry, Engineering Polytechnic School, Universidade de Santiago de Compostela, Lugo 27002, Spain

^2^Department of Plant Biology and Soil Science, Faculty of Sciences, Campus Ourense, University of Vigo, Ourense 32004, Spain

*** Correspondence:** Avelino Núñez-Delgado: avelino.nunez@usc.es

# Additional information regarding materials

The forest soil (developed over granite) was sampled near an aluminum facility (San Cibrao, Lugo, Spain). The vineyard soil samples were taken in Sober (Lugo, Spain). The pyritic material was sampled in a mine tailing (Touro, A Coruña, Spain). The granitic material was sampled in Santa Cristina (Ribadavia, Ourense, Spain). For both soils, pyritic material and granitic material, samples were taken at 0-20 cm depth by means of a soil auger, performing composite samples by mixing 10 subsamples in each case. The fine and coarse mussel shells were sampled at Abonomar S.L. (A Illa de Arousa, Pontevedra, Spain). Mussel shell ashes were from Calizamar S.L. (Boiro, A Coruña, Spain). Oak oven wood ash was sampled in Baleira (Lugo, Spain). The slate processing fines were from Europizarras S.L. (A Fonsagrada, Lugo, Spain). For all these materials, composite samples were also performed by mixing 10 subsamples in each case. Pine sawdust was form Vitakraft (sold in the market), and a composite sample was performed by mixing 10 subsamples taken from the commercial packages previously obtained in the market. Figure S1 shows maps situating the places where the different samples were taken, all of them in Galicia (NW Spain).

In the laboratory, these materials were air dried, sieved (through 2 mm), and subjected to chemical determinations and trials (carried out on the <2 mm fraction), with triplicate determinations for all materials.

**Characterization of materials**

C and N were quantitatively determined as per Chatterjee et al. (2009) on triplicate 5-g samples using an auto-analyzer (Tru Spec CHNS, LECO corporation, USA). pH in water, using 10 g of solid sample, with solid:liquid relation 1:2.5, and a pH-meter (model 2001, Crison, Spain) (McLean, 1982). Exchangeable Ca, Mg, Al, Na and K were determined as per Sumner and Miller (1996), after displacement from 5-g samples with a NH_4_Cl 1 M solution, then quantifying Na and K by atomic emission spectroscopy, and also Ca, Mg and Al (atomic absorption spectroscopy) (AAnalyst 200, Perkin Elmer, USA); the effective cationic exchange capacity (eCEC) was calculated as the sum of these cations (Kamprath, 1970). 1-g samples were used to determine total concentration of P, using nitric acid (65%) digestion (microwave assisted), followed by UV-visible spectroscopy (UV-1201, Shimadzu, Japan) (Tan, 1996). 1-g samples were used to determine total concentrations of Na, K, Ca, Mg, Al, Fe, Mn, As, Cd, Cr, Cu, Ni and Zn, using nitric acid (65%) digestion (microwave assisted), and ICP-mass quantification (820-NS, Varian, USA) (Nóbrega et al., 2012). Finally, 1-g samples were used to determine Al_o_ and Fe_o_ (total non-crystalline Al and Fe), using ammonium oxalate solutions extractions, and atomic absorption spectroscopy (by means of AAnalyst 200, Perkin Elmer, USA) (Álvarez et al., 2012). All trials were performed by triplicate.


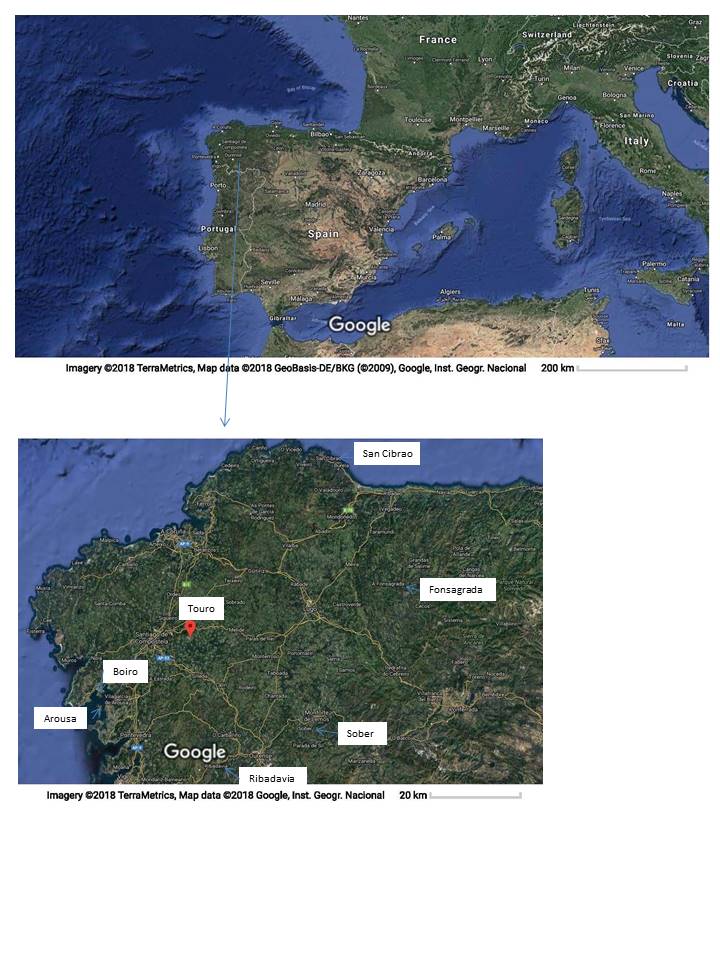
Figure S1. Maps showing the places where the different soils, wastes and by-products were sampled

**Table S1. General characteristics of the solid materials (average values for 3 replicates, with coefficients of variation always <5%)**

|  | Forest | Vineyard | Pyritic | Granitic | Oak |
| --- | --- | --- | --- | --- | --- |
|  | soil | soil | material | material | ash |
| C (%) | 4.22 | 2.94 | 0.26 | 0.11 | 6.69 |
| N (%) | 0.33 | 0.23 | 0.04 | 0.04 | 0.10 |
| pH_water_ | 5.65 | 4.48 | 2.97 | 5.72 | 11.19 |
| Ca_e_ (cmol_(+)_ kg^-1^) | 4.37 | 1.78 | 0.36 | 0.18 | 9.81 |
| Mg_e_ (cmol_(+)_ kg^-1^) | 0.66 | 0.24 | 0.29 | 0.13 | 8.49 |
| Na_e_ (cmol_(+)_ kg^-1^) | 0.33 | 0.14 | 0.14 | 0.27 | 20.53 |
| K_e_ (cmol_(+)_ kg^-1^) | 0.60 | 0.83 | 0.24 | 0.31 | 152.44 |
| Al_e_ (cmol_(+)_ kg^-1^) | 1.92 | 2.28 | 2.86 | 1.63 | 0.00 |
| eCEC (cmol_(+)_ kg^-1^) | 7.88 | 5.27 | 3.89 | 2.53 | 191.2 |
| P_T_ (mg kg-1) | 423.9 | 679.3 | 606.3 | 88.62 | 663.7 |
| Ca_T_ (mg kg^-1^) | 708.5 | 607.1 | 603 | <0.01 | 81031 |
| Mg_T_ (mg kg^-1^) | 830.5 | 5003 | 8384 | 355 | 24505 |
| Na_T_ (mg kg^-1^) | 515.1 | 297.6 | 412 | 102 | 8095 |
| K_T_ (mg kg^-1^) | 1544 | 5441 | 3186 | 1434 | 70661 |
| As_T_ (mg kg^-1^) | 4.18 | 3.41 | 7 | 3 | 4.00 |
| Cd_T_ (mg kg^-1^) | 0.43 | 0.14 | 0.08 | <0.001 | 0.18 |
| Cr_T_ (mg kg^-1^) | 18.35 | 41.44 | 99 | 3 | 66.67 |
| Cu_T_ (mg kg^-1^) | 15.72 | 521.1 | 773 | 7 | 590.5 |
| Ni_T_ (mg kg^-1^) | 10.69 | 21.73 | 5 | 1 | 51.51 |
| Zn_T_ (mg kg^-1^) | 36.74 | 49.57 | 58 | 18 | 728.9 |
| Mn_T_ (mg kg^-1^) | 92.99 | 305.4 | 296 | 24 | 6778 |
| Al_T_  (mg kg^-1^) | 22676 | 25664 | 9624 | 5981 | 20218 |
| Fe_T_ (mg kg^-1^) | 9486 | 21284 | 135157 | 3505 | 41425 |
| Al_o_ (mg kg^-1^) | 4275 | 2003 | 563 | 1425 | 8722 |
| Fe_o_ (mg kg^-1^) | 2333 | 1239 | 41860 | 224 | 5239 |

**X_e:_ exchangeable concentration of the element; X_T_: total concentration of the element; Al_o_, Fe_o_: extracted with ammonium oxalate**

**Table 1 (Continuation)**

|  | Shell | Fine | Coarse | Pine- | Slate | |
| --- | --- | --- | --- | --- | --- | --- |
|  | ash | shell | shell | sawdust | fines | |
| C (%) | 13.21 | 11.43 | 12.67 | 46.13 | 0.2 |  |
| N (%) | 1.13 | 0.21 | 0.36 | 0.03 | 0.02 |  |
| pH_water_ | 12.54 | 9.39 | 9.11 | 4.91 | 8.61 |  |
| Ca_e_ (cmol_(+)_ kg^-1^) | 39.27 | 24.75 | 12.64 | 5.39 | 4.31 |  |
| Mg_e_ (cmol_(+)_ kg^-1^) | 7.47 | 0.72 | 0.58 | 1.37 | 0.31 |  |
| Na_e_ (cmol_(+)_ kg^-1^) | 19.92 | 4.37 | 5.24 | 0.66 | 0.63 |  |
| K_e_ (cmol_(+)_ kg^-1^) | 2.61 | 0.38 | 0.31 | 1.55 | 0.31 |  |
| Al_e_ (cmol_(+)_ kg^-1^) | 0 | 0.03 | 0.04 | 0.05 | 0.01 |  |
| eCEC (cmol_(+)_ kg^-1^) | 69.28 | 30.25 | 18.82 | 9.02 | 5.57 |  |
| P_T_ (mg kg^-1^) | 1617 | 101.5 | 186.5 | 88.04 | 661.3 |  |
| Ca_T_ (mg kg^-1^) | 247859 | 280168 | 298085 | 8088 | 2810 |  |
| Mg_T_ (mg kg^-1^) | 5286 | 980.6 | 1020 | 164.4 | 11797 |  |
| Na_T_ (mg kg^-1^) | 8074 | 5173 | 5508 | 98.35 | 53.72 |  |
| K_T_ (mg kg^-1^) | 896 | 202.1 | 80.57 | 540.7 | 991.3 |  |
| As_T_ (mg kg^-1^) | 1.71 | 1.12 | 0.48 | 0.39 | 3.1 |  |
| Cd_T_ (mg kg^-1^) | 63.09 | 0.07 | 0.02 | 50.82 | 95.18 |  |
| Cr_T_ (mg kg^-1^) | 4596 | 4.51 | 1.32 | 234.2 | 54010 |  |
| Cu_T_ (mg kg^-1^) | 31.75 | 6.72 | 3.20 | 14.87 | 30.95 |  |
| Ni_T_ (mg kg^-1^) | 3421 | 8.16 | 5.64 | 260.6 | 24737 |  |
| Zn_T_ (mg kg^-1^) | 18.75 | 7.66 | 7.71 | 0 | 36.89 |  |
| Mn_T_ (mg kg^-1^) | 18.67 | 33.75 | 5.70 | 5.19 | 28.46 |  |
| Al_T_ (mg kg^-1^) | 3421 | 433.2 | 93.89 | 260.7 | 24737 |  |
| Fe_T_ (mg kg^-1^) | 4596 | 1855 | 170.37 | 234.2 | 54010 |  |
| Al_o_ (mg kg^-1^) | 1733 | 178.3 | 85.00 | 112.5 | 730.6 |  |
| Fe_o_ (mg kg^-1^) | 1659 | 171.0 | 42.67 | 15.62 | 1256 |  |

**X_e:_ exchangeable concentration of the element; X_T_: total concentration of the element; Al_o_, Fe_o_: extracted with ammonium oxalate**

**References**

Álvarez E, Fernández-Sanjurjo MJ, Núñez A, Seco N, Corti G 2012 Aluminium fractionation and speciation in bulk and rhizosphere of a grass soil amended with mussel shells or lime. Geoderma 173/174:322–329

Chatterjee A, Lal R, Wielopolski L, Martin MZ, Ebinger MH 2009 Evaluation of Different Soil Carbon Determination Methods. Cr Rev Plant Sci 28:164–178

Kamprath EJ 1970 Exchangeable aluminium as a criterion for liming leached mineral soils. Soil Sci Soc Am P 34:252–54

McLean EO 1982 Soil pH and Lime Requirement. In: Methods of Soil Analysis Part 2 Chemical and Microbiological Properties. ASA, Madison, pp 199–223

Nóbrega JA, Pirola C, Fialho LL, Rota G, de Campos CE, Pollo F 2012 Microwave-assisted digestion of organic samples: How simple can it become? Talanta 98:272–276

Sumner ME, Miller WP 1996 Cation exchange capacity and exchange coefficients. In: Methods of Soil Analysis Part 3 Chemical Methods. ASA, Madison, pp 437–474

Tan KH 1996 Soil sampling, preparation, and analysis. Marcel Dekker, New York

**
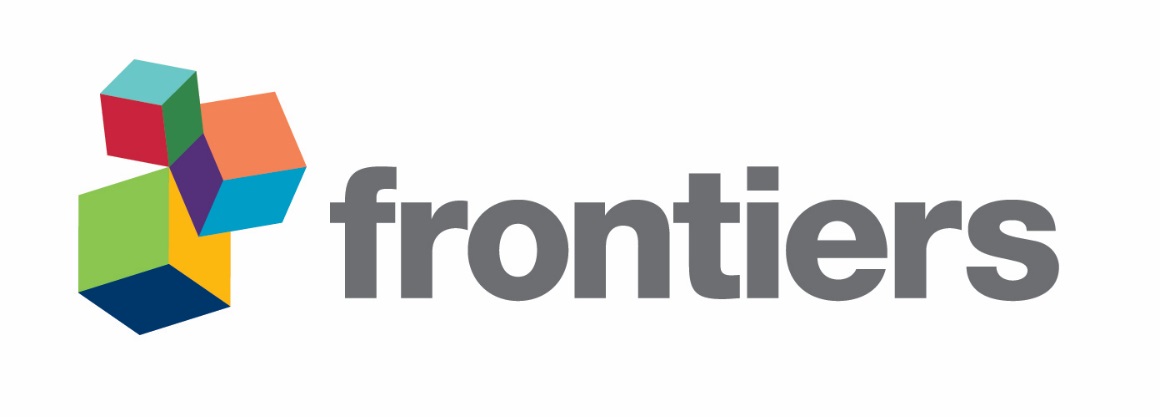
**
